# Traffic light at DSB–transit regulation between gene transcription and DNA repair

**DOI:** 10.1002/1873-3468.15024

**Published:** 2024-09-27

**Authors:** Stefania Modafferi, Francesca Esposito, Sara Tavella, Ubaldo Gioia, Sofia Francia

**Affiliations:** ^1^ Istituto di Genetica Molecolare “Luigi Luca Cavalli Sforza”– Consiglio Nazionale delle Ricerche Pavia Italy; ^2^ PhD Program in Biomolecular Sciences and Biotechnology (SBB) Istituto Universitario di Studi Superiori (IUSS) Pavia Italy; ^3^ PhD Program in Genetics, Molecular and Cellular Biology (GMCB) University of Pavia Pavia Italy; ^4^ IFOM‐ETS – The AIRC Institute of Molecular Oncology Milan Italy

**Keywords:** DNA damage response, DNA double strand break, DNA repair, non‐coding RNAs, transcription

## Abstract

Transcription of actively expressed genes is dampened for kilobases around DNA lesions via chromatin modifications. This is believed to favour repair and prevent genome instability. Nonetheless, mounting evidence suggests that transcription may be induced by DNA breakage, resulting in the local *de novo* synthesis of non‐coding RNAs (ncRNAs). Such transcripts have been proposed to play important functions in both DNA damage signalling and repair. Here, we review the recently identified mechanistic details of transcriptional silencing at damaged chromatin, highlighting how post‐translational histone modifications can also be modulated by the local synthesis of DNA damage‐induced ncRNAs. Finally, we envision that these entangled transcriptional events at DNA breakages can be targeted to modulate DNA repair, with potential implications for locus‐specific therapeutic strategies.

## Graphical abstract

Upon the generation of a break, DNA damage response activation represses transcription of genes *in cis* via an ATM‐ and PRC1‐dependent chromatin modification and compaction. Concomitantly, RNAPII accumulates in proximity to DNA ends and locally synthesizes chromatin‐bound ncRNAs. The two events might be functionally linked: damage‐induced ncRNAs are possibly key players recruiting the PRC1 subunit BMI1 to the lesion leading to chromatin ubiquitination and transcriptional repression.

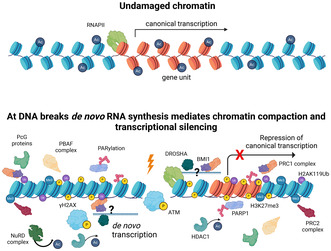

## Abbreviations

**ASOs**, antisense oligonucleotides

**DDR**, DNA damage response

**DDRNA**, DNA damage response RNA

**dilncRNA**, damage‐induced long non‐coding RNA

**DISC**, damage induced transcriptional silencing in cis

**DSB**, DNA double strand break

**lncRNA**, long non‐coding RNA

**ncRNA**, non‐coding RNA

**PTM**, post‐translational modifications

**tDDRNA**, telomeric DNA damage response RNA

## DNA damage‐induced transcriptional silencing

Transcriptional repression of genes *in cis* to Double Strand Breaks (DSB) is a well‐documented event occurring upon DNA breakage [1, 2]. This finely tuned phenomenon has been referred to as Damage Induced transcriptional Silencing *in Cis* (DISC) [3]. DISC is dependent on the activation of DNA damage response (DDR) apical kinases, Ataxia‐telangiectasia mutated (ATM) [1, 3] but also DNA‐dependent protein kinase, catalytic subunit (DNA‐PKcs) [4], as demonstrated by the fact that the inhibition of their kinase activity allows transcriptional recovery even in the presence of DNA breaks [1, 4, 5]. Thus, the dampening in transcription of a damaged gene is, surprisingly, not due to the physical presence of breakages on the DNA, but rather is a process actively regulated via chromatin modification. Indeed, transcriptional silencing is not restricted to the single gene carrying the break but involves several genes in the surroundings of the DSB, reaching distances of hundreds of kilobases [1, 3]. Histone post‐translational modifications (PTMs) have a prominent role in chromatin regulation and indeed also DISC is achieved by extensive chromatin modification via ubiquitination, methylation, and deacetylation, which results in chromatin compaction and consequent stalling or eviction of the elongating RNA‐Polymerase II (RNAPII) [3, 4, 6, 7].

**Fig. 1:**
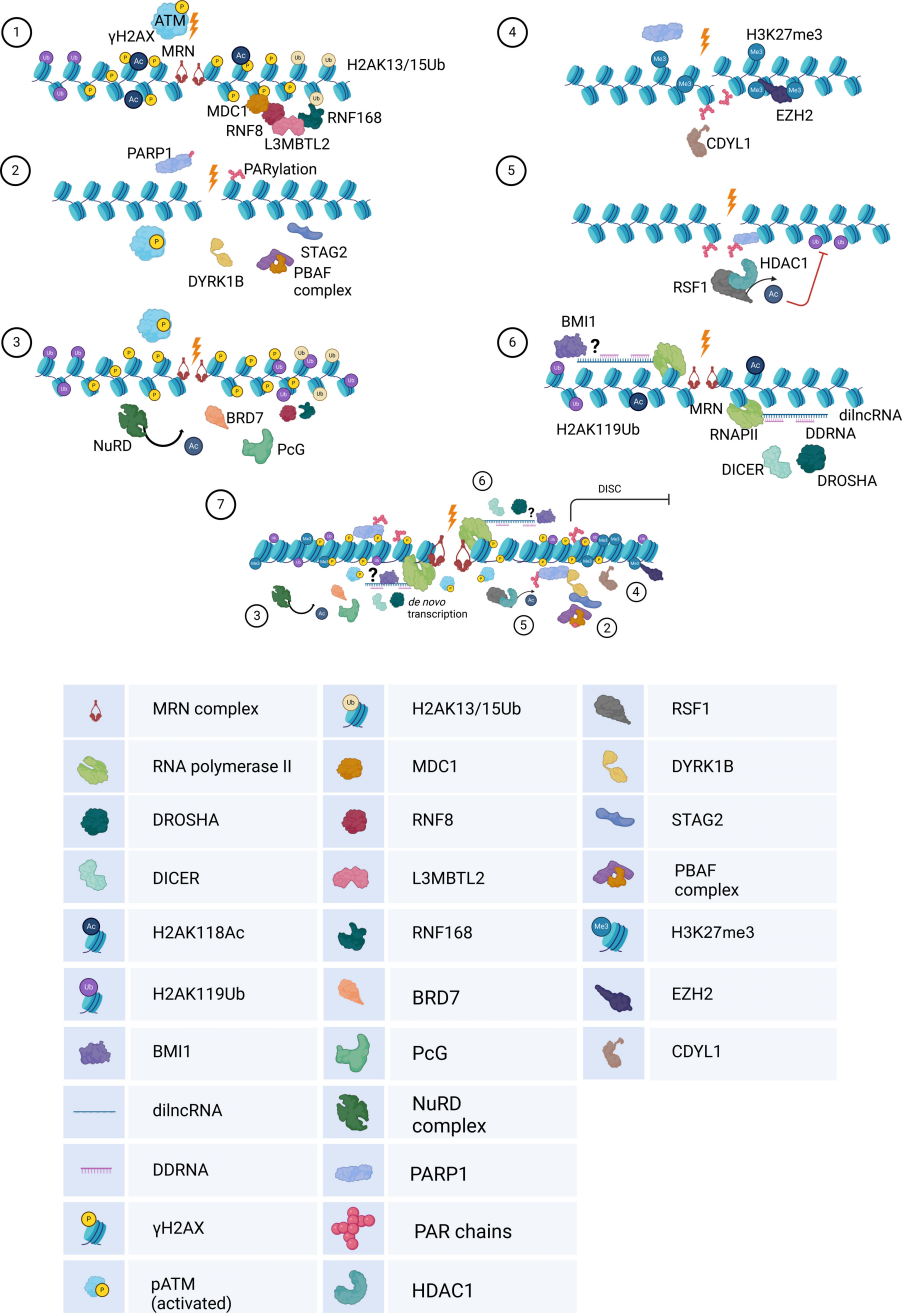
DNA damage response (DDR) activation and Damage Induced Transcriptional Silencing *in Cis* (DISC) is induced at DNA double strand breaks by multiple redundant signalling pathways and coexist with *de novo* synthesis of damaged induced ncRNAs. Top panel: (1) Upon the generation of a double strand break (lightning), the sensor complex MRN recruits ATM to the break site [3]. ATM phosphorylates the histone H2AX on Ser139 (known as γH2AX), promoting he recruitment of the mediator factor MDC1 [10], and the E3 ubiquitin ligase RNF8, which ubiquitinates the Polycomb factor L3MBTL2 [11], anchoring the E3 ubiquitin ligase RNF168. This second Ubiquitin ligase deposits H2AK13/15Ub histone marks required for DNA repair factors [12]. (2) Furthermore, ATM and Poly [ADP‐ribose] polymerase 1 (PARP1 – another apical DDR factor) recruits DYRK1B to DSB. This kinase in turn recruits PBAF and STAG2 cohesin complexes to break sites, leading to chromatin compaction and gene silencing [28, 32]. (3) ATM also promotes the recruitment of the Bromodomain containing protein BRD7 to DSBs which in turn controls the activity of the chromatin‐modifying complexes Polycomb group 1 and 2 (PcG) and Nucleosome Remodelling and De‐Acetylase (NuRD) complexes, depositing in proximity to DNA damage repressive histone marks on chromatin [31]. (4) PARP1‐dependent recruitment of CDYL1 allow the association of the PRC2 subunit EZH2 to DSBs, mediating the deposition of the repressive H3K27me3 histone mark at the damaged chromatin [33]. (5) PARP1 also contributes to DISC by recruiting remodelling and splicing factor RSF1 and HDAC1, which deacetylate histones, leading to chromatin compaction and silencing [37]. (6) Following the generation of a double strand break, RNAPII synthesizes dilncRNAs that can be then processed by DROSHA and DICER into shorter DDRNAs, necessary to foster DDR signalling [43, 46]. Polycomb repressive complex 1 (PRC1) composed by the E3 catalytic subunit RING1A/B and the Ring finger factor BMI1, instead is known to monoubiquitinate H2A on K119, a repressive modification leading to chromatin compaction and transcriptional repression of DNA break flanking genes [22]. In our preprint, we present data suggesting that the generation of dilncRNAs and DDRNAs might be required for the recruitment of BMI1 and transcriptional silencing at DSB. (7) Hypothetical working model showing how the different factors showed in panels 2–6 contribute to DISC at the same damaged locus. Small numbers refer to panels 2–6 described above. We propose that a dual modulation of transcription exists at DSBs. On the one hand, in the vicinity of the break, non‐coding RNAs are *de novo* synthesized by RNAPII and further processed by DROSHA and DICER, especially at repetitive loci. On the other hand, the chromatin regions surrounding the break are compacted by histone post‐translational modifications. We propose that these two apparently opposite events might be functionally linked and that histone modifiers might be also recruited at damaged chromatin by sequence specific non‐coding RNAs generated upon DNA damage. Bottom panel: table indicating each factor involved in DDR, DISC and *de novo* synthesis. ? indicates a postulated interaction between BMI1 and dilncRNAs reported in our pre‐print discussed in the text but still unpublished on peer‐reviewed journal. The figure was created with BioRender.com.

Upon generation of a DSB, the lesion is recognized by the sensor complex MRE11‐RAD50‐NBS1 (MRN) which in turn recruits ATM [8, 9]. Following ATM activation, histone H2AX is phosphorylated on Ser139 (γH2AX). The formation of γH2AX enables the recruitment of downstream DDR factors such as MDC1 [10]. Lethal malignant brain tumour‐like protein 2 (L3MBTL2) is recruited at DSB by MDC1 and orchestrates the recruitments of the RING‐type E3 ubiquitin ligases RNF8 and RNF168 [11]. RNF8 ubiquitylates L3MBTL2, which in turn facilitates the recruitment of RNF168. It is in fact RNF168, and not RNF8, that catalyses the mono‐ubiquitination of the histones H2A and H2AX specifically on Lys 13–15 (H2AK13,15Ub) [12], which stimulates DNA repair through BRCA1 and 53BP1 recruitment (Fig. 1 panel 1 and Table 1).

**Table 1:** List of factors involved in the DDR and their functions.

Factor	Function in the DDR	References
Ataxia telangectasia mutated (ATM)	Apical kinase of the DDR pathway. Phosphorylates H2AX on Ser 139 (γH2AX) to initiate DDR signalling.	[8, 9]
Bromodomain containing 7 (BRD7)	Subunit of the Polybromo‐Associated BAF (PBAF) chromatin remodelling complex. Recruited by ATM. Allows recruitment of several DDR factors including the MRN complex, BRCA1, 53BP1 and RNF168 to DNA lesions.	[31]
DICER	Required for secondary recruitment of DDR factors.	[45, 46]
DNA damage RNAs	Favour DNA repair by HR and NHEJ by promoting secondary recruitment of DDR factors.	[45, 46]
DNA‐dependent protein kinase catalytic subunit (DNA‐PKcs)	Apical kinase of the DDR and repair pathways.	[4, 8]
DROSHA	Required for secondary recruitment of DDR factors.	[45, 46]
Histone methyl‐lysine binding protein 2 (L3MBTL2)	Polycomb group proteins (PcG) member. Recruited by interaction with MDC1. Ubiquitylated by RNF8 to facilitate recruitment of RNF168 at the lesion.	[11, 18]
Mediator of DNA damage checkpoint 1 (MDC1)	Scaffold protein. Recruited at DSB via interaction with γH2AX. Acts as a scaffold to recruit RNF8.	[10]
MRE11‐RAD50‐NBS1 (MRN) complex	Senses DNA double strand breaks and recruits ATM and DROSHA.	[8, 9, 43]
Ring finger protein 168 (RNF168)	RING‐type E3 ubiquitin ligase. Ubiquitylates H2A and H2AX on Lys 13‐15, allowing DNA repair via BRCA1 and 53BP1 recruitment.	[11]
Ring finger protein 8 (RNF8)	RING‐type E3 ubiquitin ligase. Ubiquitylates L3MBTL2 to start the signalling cascade that results in 53BP1 presence at the break site.	[11]
RNA polymerase III (RNAPIII)	Catalyses the synthesis of ncRNAs that form hybrids with the DNA template. Formation of these hybrids stimulates HR repair.	[40]
Single‐stranded DNA‐damage‐associated small RNAs (sdRNAs)	Synthesised by RNAPII in chromatin regions that are prone to form R‐loops. Mediate the assembly of BRCA1 repair complex and are required for efficient resolution of DNA single‐strand breaks (SSBs).	[51]

In most genomic loci, the maintenance of facultative heterochromatin and the consequential repression of transcription, is dependent on the activity of the Polycomb group proteins (PcGs), differentiated into two large repressive complexes, namely Polycomb Repressive Complex 1 (PRC1) and Polycomb Repressive Complex 2 (PRC2) [13]. The starting modification in transcriptional repression is the mono‐ubiquitination of histone H2A at lysine 119 (H2AK119ub1), catalysed by the PRC1 complex composed by the core catalytic subunits, the E3 ubiquitin ligase RING1A/B, in cooperation with Polycomb Group RING finger protein 4 (PCGF4 or BMI1) [14]. This modification can in turn promote the PRC2‐dependent methylation of histone H3 on lysine 27 via Enhancer of Zeste Homolog 1 or 2 (EZH1 or EZH2) activity [14]. Both complexes contribute to the correct organism development by establishing and maintaining cell type‐specific transcriptional programs [15]. Also, L3MBTL2 belongs to the PcG family and interacts with the histone deacetylase domain of histone deacetylase 3 (HDAC3), thus negatively regulating transcription of target genes [16]. Importantly, L3MBTL2 has been implicated in transcriptional repression and chromatin compaction via PRC1 [17, 18]. In addition, BMI1/RING1b‐dependent H2AK119 ubiquitination has already been shown to functionally promote Non‐Homologous End Joining (NHEJ) in the context of deprotected telomeres and favour repair of ionizing radiation (IR)‐induced DSBs at heterochromatin loci [19]. Although initially the recruitment of the BMI1‐PRC1 complex at individual endogenous DSBs was controversial [20], restricting its role to clusters of DSBs, recently it has been confirmed that the BMI1‐PRC1 complex catalyses H2AK119ub1 deposition also at one separate DSB to promote DISC [21]. Importantly, we also show in our preprint that BMI1 is recruited at endogenous DSBs generated by inducible restriction enzymes and by CRISPR/Cas9‐mediated cleavage [22]. Recently, BMI1‐deposited H2AK119ub1 has been shown to concomitantly inhibit RNAPII transcription and recruit to DSBs the CtIP factor, which supports resection in S‐G2 cells, initiating repair via the Homologous Recombination (HR) pathway [23]. Nevertheless, H2AK119ub1 has also been involved by different studies in NHEJ in G1 cells [24, 25]. Thus, BMI1 seems to foster DSBs repair via both HR and NHEJ. At UV‐induced lesions, BMI1 has been shown to recruit the Ubiquitin Protein Ligase E3 Component N‐Recognin 5 (UBR5) that negatively regulates the function of the Facilitates Chromatin Transcription (FACT) histone chaperone complex [6]. A canonical function of FACT is to facilitate the nucleosomal reorganization to favour transcriptional elongation of RNAPII [26]. The ubiquitination by UBR5 transiently inhibits FACT and consequently RNAPII elongation to promote DNA repair [6].

At DSBs, UBR5 is retained by its interaction with the Ovarian Tumour Deubiquitinase 5 (OTUD5) [27]. The OTUD5‐UBR5 axis contributes to DISC by preventing access of elongating RNAPII to break‐bearing genes. Independently from its interaction with UBR5, OTUD5 also interacts with SPT16, another component of the FACT complex. In this way, FACT activity is inhibited also *in cis* to DSBs and prevents RNAPII access to damaged chromatin [27]. In the past years, other reports suggested that DNA‐PKcs activation upon DNA damage in actively transcribed genes, locally induces RNAPII ubiquitination and its proteasomal degradation by promoting the interaction of RNAPII with the HECT E3 ubiquitin ligase WWP2 (WW Domain Containing E3 Ubiquitin Protein Ligase 2) [4, 5]. Thus, despite most studies suggest that DISC inhibits RNAPII elongation, some studies proposed that a fraction of RNAPII is actively removed from the damaged chromatin by targeting it to proteasomal degradation, even if persistent DNA damage seems to be required to activate this phenomenon [5].

Moreover, the serine/threonine kinase, Dual Specificity Tyrosine Phosphorylation Regulated Kinase 1B (DYRK1B) has been recently identified as a new key factor in DISC, working downstream of ATM and shutting down transcription in the presence of DSBs. DYRK1B interacts with the Euchromatic Histone Lysine Methyltransferase 2 (EHMT2) [28], a methyltransferase that methylates Lysine 9 in Histone H3 (a common marker of heterochromatin), leading to transcriptional repression, upon DSBs generated by radiation in mammalian cells [29, 30]. Similarly to ATM inhibition, loss of DYRK1B activity restores transcription, even if the presence of DSBs persists. Another interactor of DYRK1B is BRD7 (Bromodomain Containing 7) [28], a subunit of the Polybromo‐Associated BAF (PBAF) chromatin remodelling complex (Fig. 1 panel 2). ATM‐mediated recruitment of BRD7 at break sites promotes DISC by engaging other chromatin repressive complexes, such as PRC2 and Nucleosome Remodelling Deacetylase (NuRD) complex (Fig. 1 panel 3) and promotes recruitment of several DDR factors, including MRN, BRCA1, 53BP1 and RNF168, to DNA lesions [31]. Similar results were obtained upon depletion of the Brahma‐Related Gene 1 (BRG1), the catalytic subunit of the PBAF complex [32].

Also, the cohesin complex – crucial in mediating sister chromatids faithful segregation in the G2 phase of the cell cycle – has been found to function in transcriptional repression. Indeed, loss of STAG2 – a cohesin subunit – abolishes DISC [32]. Of note, this novel function of cohesin is not restricted to the G2 phase, but instead occurs throughout the cell cycle and is independent from its role during mitosis [32]. Interestingly, loss of DYRK1B impairs STAG2 recruitment to laser tracks [28], suggesting that DYRK1B may act upstream of the cohesin complex in modulating DISC (Fig. 1 panel 2).

PARP1 (poly‐ADP‐ribose polymerase 1) also plays a key role in DISC by mediating the recruitment of several factors that control gene silencing. Recent evidence shows that the FBXL10‐RNF68‐RNF2 (FRRUC) ubiquitin ligase complex is recruited to DNA lesions in a PARP1‐dependent manner and mediates BMI1 recruitment and H2AK119 ubiquitination at break sites [7]. PARP1 also modulates the recruitment of the chromatin reader Chromodomain Y like 1 (CDYL1) to DSBs, which in turn favours the PRC2 subunit EZH2 assembly at DSBs (Fig. 1 panel 4). EZH2 then catalyses H3K27me3 deposition, inducing DISC [33, 34]. Finally, also the Remodelling and Spacing Factor 1 (RSF1), a component of the chromatin assembly complex RSF [35], regulates transcription [36] by promoting HDAC1 association to DSBs, to remove the H2AK118ac modification (Fig. 1 panel 5), which inhibits H2AK119ub1 deposition and DNA repair [37]. For a schematic overview of the factors involved in DISC see Table 2.

**Table 2:** List of factors involved in DISC and their functions.

Factor	Function in DISC	References
Ataxia telangectasia mutated (ATM)	Apical kinase of the DISC pathway. Required for the recruitment of many downstream factors and chromatin‐remodelling complexes that silence canonical transcription upon double strand break (DSB) formation. Phosphorylates H2AX on Ser 139 (γH2AX) to initiate DISC signalling.	[1, 3, 8, 9]
	Allows BMI1‐PRC1‐dependent H2AK119Ub1 deposition.	[21]
BMI1	Acts in concert with PRC1 to deposit H2AK119Ub1.	[14, 21]
	At UV‐induced lesions, recruits UBR5 to sites of damage to inhibit FACT activity.	[6]
	Favour repair of lesions generated by ionizing radiation at heterochromatic loci.	[19]
Bromodomain containing 7 (BRD7)	Subunit of the Polybromo‐Associated BAF (PBAF) chromatin remodelling complex.	[28]
	Interacts with ATM and DYRK1B.	[28, 31]
	At DSBs, it engages PRC2 and the Nucleosome Remodelling Deacetylase (NuRD) complexes to compact chromatin.	[28, 31]
Chromodomain Y like 1 (CDYL1)	Chromatin reader. Recruited to DSB in a PARP1‐dependent manner where it favours PRC2 assembly.	[32, 33]
Cohesin complex	Functions in transcriptional repression.	[32]
	Possibly recruited by DYRK1B.	[28]
Damage‐induced long non‐coding RNAs (dilncRNAs)/DNA damage RNAs (DDRNAs)	Possibly required for BMI1 recruitment at break sites.	[22]
DICER	Recruits BMI1 at break sites.	[22]
DNA‐dependent protein kinase catalytic subunit (DNA‐PKcs)	Apical kinase of the DISC pathway.	[4]
	Together with WWP2, induces RNAPII proteasomal degradation when DNA damage occurs at actively transcribed loci.	[4, 5]
DROSHA	Recruits BMI1 at break sites.	[22]
Dual specificity tyrosine phosphorylation regulated kinase 1B (DYRK1B)	Acts downstream of ATM and PARP1. Recruits EHMT2, PBAF and possibly cohesin complexes to DSBs.	[28]
Euchromatic histone lysine methyltransferase 2 (EHMT2)	Methylates H3 on Lys 9 (heterochromatic marker).	[29, 30]
Facilitates chromatin transcription (FACT) histone chaperone complex	Nucleosomal reorganization to favour RNAPII transcription elongation.	[26]
FBXL10‐RNF68‐RNF2 (FRRUC) ubiquitin ligase complex	Recruited to DNA lesion in a PARP1‐dependent fashion. Mediates BMI1 recruitment and H2AK119Ub1 deposition at break sites.	[7]
Histone methyl‐lysine binding protein 2 (L3MBTL2)	Polycomb group proteins (PcG) member.	[16]
	Promotes gene silencing and chromatin compaction together with HDAC3 and PRC1.	[16, 17, 18]
Ovarian tumour deubiquitinase 5 (OTUD5)	Stabilizes UBR5 at DSBs. Interacts *in cis* with FACT subunit SPT16 to further prevent RNAPII elongation.	[27]
Poly(ADP‐ribose) polymerase 1 (PARP1)	Recruits several DISC factors.	[7]
Polycomb repressive complex 1 (PRC1)	H2AK119Ub1 deposition.	[13, 14]
Polycomb repressive complex 2 (PRC2)	H3K27me3 deposition.	[13, 14]
Remodelling and spacing factor 1 (RSF1)	Component of the chromatin assembly complex RSF.	[35]
	Promotes HDAC1 association to DSB1 to remove H2AK118Ac.	[36, 37]
Ubiquitin protein ligase E3 component N‐recognin 5 (UBR5)	Ubiquitylation of FACT with inhibitory outcomes.	[6]
WWP2 (WW domain containing E3 ubiquitin protein ligase 2)	Induces RNAPII proteasomal degradation when DNA damage occurs at actively transcribed loci.	[5]

By dampening pre‐existing transcription upon DNA damage generation, all these mechanisms cooperate to allow a switch between genome expression and its repair. Of note, unperturbed transcription in the presence of DSBs is associated with persistent DNA damage, increased chromosome aberrations and genome instability [7, 28, 31, 32, 33], further strengthening the intimate relationship between DISC and proper DSB repair.

## Biogenesis and functions of non‐coding RNAs at damaged chromatin

**Fig. 2:**
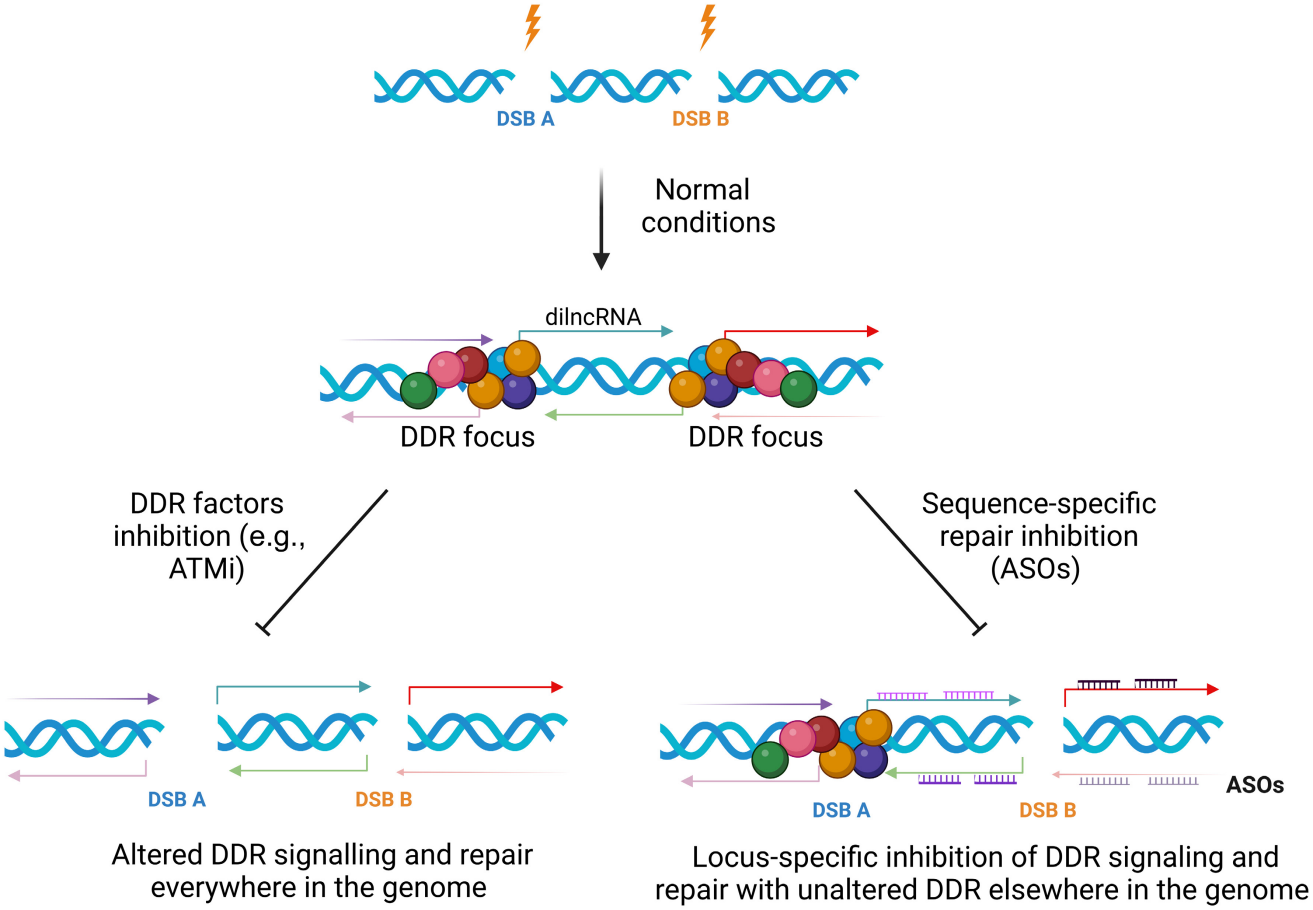
Site‐specific DDR modulation via antisense oligonucleotides (ASOs). Upon generation of a DNA double strand break (DSB) (lightning), damage‐induced long non‐coding RNAs (dilncRNAs) are transcribed from the break, stimulating the recruitment or the activation of DNA damage response (DDR) factors and the formation of a DDR focus at the site of damage. Inhibitors targeting the activity of DDR factors, such as ATM inhibitors (ATMi, bottom left panel), simultaneously switches off the DDR signalling of all DSBs present in the genome. In contrast, the treatment with site‐specific antisense oligonucleotides (ASOs, bottom right panel) targeting dilncRNAs, might inhibit DDR in a sequence‐specific manner. The figure was created with BioRender.com.

As described above, pre‐existing transcription is halted in response to local DNA damage [1, 3, 38, 39]. However, various evidence suggest that *de novo* synthesis of RNA is induced at sites of DNA damage [40, 41, 42, 43, 44]. Why should cells initiate a transcription process, which is both energetically and materially demanding, when their genetic products are at risk of being irremediably compromised by a genotoxic insult? Actually, and more intriguingly, nascent transcripts generated at the damaged chromatin have been proposed to facilitate both the detection and repair of the DNA lesion, ultimately improving cell's ability to preserve genome integrity. For instance, it has been shown that the formation of a DSB is sufficient to recruit RNAPII, Mediator and different components of the Pre‐Initiation Complex (PIC), which drives the synthesis of long non‐coding RNAs (lncRNAs), hence called damage‐induced lncRNAs (dilncRNAs) [44]. These transcripts, normally few hundreds bases long, are not abundantly expressed and are largely retained chromatin‐bound. Nevertheless, dilncRNAs can be further processed by DROSHA and DICER to generate smaller molecules, named DNA Damage response RNAs (DDRNAs) (Fig. 1 panel 6), that, via base‐pairing with their longer precursors, cooperate in the gathering of DDR and repair factors at the sites of damage [43, 44, 45, 46]. In particular, these ncRNAs, are believed to promote the assembly at DSBs of 53BP1 foci, an important factor involved in DNA damage repair by NHEJ and induce a liquid–liquid phase separation (LLPS) in these 53BP1:RNA complexes [44]. Interestingly, sequence‐specific disruption of dilncRNA/DDRNA functions, through sequence specific antisense oligonucleotides (ASOs) (Fig. 2), impact on DDR signalling and LLPS of 53BP1 foci, a fact that has been shown to contrast the resolution of DSBs as measured by comet assay [42, 43, 44, 45, 46]. Conversely, pharmacological treatment with enoxacin, a small molecule that enhances DICER activity [47], can stimulate DDRNA production, and favours a faster and a more accurate DSB repair by NHEJ [48, 49, 50]. Small ncRNAs have also been observed to be generated by RNAPII in proximity to chromatin regions that are prone to form R‐loop structures [51]. Such molecules, named single‐stranded DNA‐damage‐associated small RNAs (sdRNAs), mediate the assembly of BRCA1 repair complex and are required for efficient resolution of DNA single‐strand breaks (SSBs) that may arise at RNAPII termination sites [51]. Interestingly, RNAPII is not alone in participating in genome protection. In fact, DSB formation can also drive RNA Polymerase III (RNAPIII) transcription [40]. RNAPIII has been found to be recruited at DSBs, whereby it catalyses the synthesis of ncRNAs that form hybrids with the DNA template [40]. Such RNA–DNA hybrids, in turn, stimulate the association of components of the HR repair machinery on the chromatin, ultimately fostering DNA damage resolution [40]. Similarly, RNAPII‐transcribed dilncRNAs, which form RNA–DNA hybrids at damaged sites during S/G2 phase, are also able to recruit BRCA1, BRCA2 and RAD51 and mediate repair by HR [52]. For a schematic list of factors involved in *de novo* transcription at DSBs see Table 3.

**Table 3:** List of factors involved in *de novo* synthesis of ncRNAs at damaged DNA and their functions.

Factor	Function in ncRNAs *de novo* synthesis	References
DICER	Processes dilncRNAs to generate shorter DNA Damage RNAs (DDRNAs).	[45, 46]
DROSHA	Processes dilncRNAs to generate shorter DNA Damage RNAs (DDRNAs).	[45, 46]
RNA polymerase II (RNAPII)	Generates damage‐induced long non‐coding RNAs (dilncRNAs) at DSBs.	[43, 44]
RNA polymerase III (RNAPIII)	Catalyses the synthesis of ncRNAs that form hybrids with the DNA template to allow HR repair.	[40]

A role for damage‐induced ncRNAs in modulating transcription at the broken chromatin has been recently proposed [22, 53]. For instance, in our preprint we showed that dilncRNAs regulate DISC and DNA repair by interacting with BMI1 upon DNA damage induction, thus promoting its association at DSBs [22]. This suggests that the newly synthetized ncRNAs act as early DNA damage responders that promote the subsequent PRC1 association at DSBs to silence pre‐existing transcription, ultimately favouring repair (Fig. 1 panel 7). Such findings further expand the range of functions played by ncRNAs generated at damaged chromatin in the maintenance of genome integrity.

## Targeting DISC at DSB to modulate DNA repair in neurodegeneration and cancer

The interdependency between DSB repair and transcriptional repression of break‐bearing and break‐flanking genes is emerging as a prominent read out of DDR activation, thus contributing to prevent genome instability and ultimately carcinogenesis onset. Several factors involved in DSB‐induced gene silencing have been shown to sustain DSB repair, via both the NHEJ or the HR pathways [54]. Indeed, factors involved in DISC are often altered in different types of tumours. For instance, the BAF180 subunit of the PBAF complex is mutated in 38% of renal clear cell carcinomas [55], EZH2 exhibits gain‐of‐function mutations in B cell lymphomas [33, 56], while the FBXL10 subunit of the FRRUC complex is found upregulated in 14% of invasive breast carcinomas [7]. Therefore, their targeting can represent an attractive pharmacological strategy in cancer therapy. As an example, cancers harbouring a specific mutation in the repressive factor STAG2 exhibit HR deficiency (HRD) [32]. This so called “BRCAness” phenotype is usually synthetically lethal to the chemotherapeutic drugs targeting the functioning of Poly‐ADP polymerases, acting upstream in the cellular response to DNA breaks, such as the PARP1 inhibitor Olaparib [57]. Therefore, the modulation of DISC may render these and other types of tumours more susceptible to PARP inhibitors, expanding treatment options or increasing their efficacy.

As mentioned above, *de novo* RNA transcription and processing at DSB sites may play a role in DISC, too [22]. Moreover, pharmacological modulation of DDRNA biogenesis through enoxacin administration, already known to enhance DNA repair efficiency [48], can stimulate DSB‐induced transcriptional silencing, representing an additional opportunity for a targeted cancer therapy. Low doses of enoxacin stimulate DDRNA production without altering miRNA levels, and boost DDR signalling resulting in a more proficient repair by NHEJ [48]. We have recently shown in our preprint that enoxacin also enhances DSB‐induced gene silencing [22], connecting DISC to the DDRNA pathway and suggesting the potential use of enoxacin to reinforce repair via NHEJ in pathological contexts. On one hand, enoxacin could be used in terminally differentiated cells, where only NHEJ pathway is accessible, to reduce cell death due to aberrant accumulation of DNA damage. For example, enoxacin treatment could promote survival of differentiated motor neurons in the context of Amyotrophic Lateral sclerosis (ALS) pathology, where growing evidence links DNA damage accumulation to FUS and TDP‐43 ALS phenotypes [58]. Indeed, enoxacin at high doses, by restoring DICER function and microRNA processing, has been shown to increase neuromuscular functions in mouse models of ALS, despite little impact on lifespan [59]. On the other hand, enoxacin could be employed in cancer therapy to shift the balance between NHEJ and HR pathways and restore NHEJ‐dependent control of unscheduled HR. In fact, resistance to PARPi can arise after a relatively short period of time in the treatment of HRD tumours due to secondary mutations in NHEJ factors that inhibit DNA end‐resection and HR. Thus, enoxacin could be used in combined therapies to boost NHEJ and restore PARPi sensitivity.

## Targeting DDRNA functions with antisense oligonucleotides

The Clustered Regularly Interspaced Short Palindromic Repeats (CRISPR)‐Cas9 system is a powerful genome‐editing tool, based on the adaptive immunity of bacteria to viruses, that is nowadays used in different applications, among which cancer treatment [60, 61]. Indeed, some CRISPR‐Cas9 approaches have been proposed to counteract cancer cells proliferation and viability by targeting chromosomal rearrangements, such as the Philadelphia chromosome found in patients affected by leukaemia [62, 63, 64, 65] or oncogenes like KRAS that, when mutated, can cause lung, colorectal and pancreatic cancers. It has been shown that Cas9, by introducing DSBs in a complementary DNA sequence, can reduce the expression of the gene products with consequent cancer cell death [66]. However, the accurate repair of Cas9‐induced DSBs can compromise the efficacy of the treatment. As introduced above, we have demonstrated that the use of ASOs targeting the dilncRNAs/DDRNAs generated upon damage inhibits the formation of a proper DDR focus and the repair of the DNA lesion (Fig. 2) [42, 43, 52, 67, 68]. ASOs are synthetic, short, single‐stranded molecules that, through Watson‐Crick base pairing, inhibit the functions of target RNAs [69]. To prove their selectivity, we took advantage of a cell line engineered to harbour the recognition site for an endonuclease flanked by arrays of the lac‐repressor binding site (Lac) and the tetracycline response element (Tet) where we monitored the efficient generation of DSBs through the detection of γH2AX foci by immunofluorescence. The treatment with ASOs against the RNAs transcribed at Tet loci upon damage revealed an impairment in DDR activation, as monitored by 53BP1 foci formation, specifically at Tet loci, while leaving DDR activation at the Lac sites within the same cell unaffected [43]. To explore the impact of DDR inactivation by ASOs on DNA repair, we exploited the use of a traffic light reporter system (TLR) to evaluate by flow‐cytometry the repair pathway choice upon DSBs induction [70]. In fact, depending on the repair mechanism employed, this system generates two different fluorescent proteins: either a functional green fluorescent protein (GFP) in case of HR or a mCherry protein in case of NHEJ [70]. ASOs against the dilncRNAs transcribed after DSBs generation caused a significant reduction of both the green and the red fluorescent signals, suggesting that ASOs can interfere with both HR and NHEJ [52].

The detection of dilncRNAs and DDRNAs synthesis upon DNA damage has been extended also to telomeres, the end of linear chromosomes [42, 67, 68]. Our group, in fact, reported that either uncapped or damaged telomeres lead to the synthesis, accumulation, and processing of telomeric dilncRNAs (tdilncRNAs) and tDDRNAs arising from telomeric DNA ends, promoting DDR proteins recruitment and telomeric DNA damage repair [42, 71]. Telomere dysfunction has been linked to cellular senescence [72], ageing [73], degenerative disorders [74] and cancer [75]. An example is the Hutchinson‐Gilford progeria syndrome (HGPS), a premature ageing disorder caused by mutations in the Lamin A (LMNA) gene, resulting in the translation of a truncated lamin A protein called progerin, with consequent chromosomal instability, telomere dysfunction and cellular senescence [67]. Our laboratory reported elevated levels of tdilncRNAs and tDDRNAs in HGPS patient fibroblasts and in an *in vivo* HGPS skin mouse model. Importantly, ASOs against tdilncRNAs and tDDRNAs molecules block DDR activation at telomeres, attenuate the detrimental progerin‐driven defects, such as senescence, and extend lifespan in HGPS mice [67]. Another context where we have delineated the role of tdilncRNAs/tDDRNAs is the alternative lengthening of telomeres (ALT) mechanism, activated by ~10–15% of cancers and characterized by telomeric damage [68]. ALT cancer cells, in fact, display higher levels of these transcripts, compared to non‐ALT cells, that are essential for their survival. In fact, inhibition of C‐rich tdilncRNAs with ASOs leads to apoptosis selectively in ALT cells [68].

Interestingly, some ASOs have already been approved and are on the market to cure human diseases. In addition, nearly 50 additional ASOs are in clinical trials for the treatment of many diseases [76]. Thus, ASOs are promising candidates as sequence‐specific DSB repair inhibitors. Excitingly, also Cas9‐based strategies are now in clinical trials to treat genetic diseases and recently the U.S. Food and Drug Administration (FDA) approved two treatments for sickle cell disease (https://www.fda.gov/news‐events/press‐announcements/fda‐approves‐first‐gene‐therapies‐treat‐patients‐sickle‐cell‐disease). Based on these results, it is tempting to speculate that in the future the combination of Cas9 and ASOs could be used as a precision cancer therapy approach. More specifically, a sgRNA could be designed to target an oncogene, such as the Philadelphia chromosome mentioned before, to introduce a sequence‐specific DSB. Upon the generation of site‐specific DNA damage by CRISPR‐Cas9 at genetic loci altered in the cancer clone, ASOs against dilncRNAs and DDRNAs could be designed to inhibit the signalling and the repair of the sole DSB generated in cancer cell, thus potentially killing them, while sparing the other cells that miss the target (Fig. 2). Importantly, in our preprint we also show that sequence‐specific ASOs can also dampen DISC [22], thus contributing in this way to inhibit restoration of the functionality of the targeted genomic locus. In the same preprint we also observe that targeting dilncRNAs reduces BMI1 association with damaged chromatin, thus allowing persistent pre‐existing gene transcription even upon DSB induction [22]. Similarly, the CRISPR‐Cas13 system can be used to target single strand RNAs [77], and in our preprint it was shown to efficiently inhibit DISC by cleaving dilncRNAs generated at a DSB in an endogenous non‐repetitive locus [22]. Thus, by inhibiting DNA repair in a cell‐specific and sequence‐specific manner, ASOs, CRISPR‐Cas9 and CRISPR‐Cas13 approaches could be implemented for killing cancer cells more and more precisely, paving the way for a new class of potent, safe and personalized cancer treatments (Fig. 2).

## Concluding remarks

In this review, we aimed at highlighting the sophisticated interplay at sites of DNA damage between canonical gene transcription, non‐coding RNAs local synthesis and chromatin modifiers, emphasizing how they coordinate each other to regulate the traffic dynamics between transcription and repair. Achieving a comprehensive understanding of these intricate processes, their mutual interactions, and regulatory mechanisms, could significantly contribute to design therapeutic intervention in various contexts, including the development of locus precision targeting in cancer and innovative therapeutic strategies for the treatment of neurodegenerative diseases.

## Author contributions

SM wrote the paragraph entitled “DNA damage‐induced transcriptional silencing” and prepared the figures and tables. UG wrote the paragraph entitled “Biogenesis and functions of non‐coding RNAs at damaged chromatin”, FE wrote the paragraph entitled “Targeting DISC at DSB to modulate DNA repair in neurodegeneration and cancer”. ST wrote the paragraph entitled “Targeting DDRNA functions with antisense oligonucleotides”. SF conceived the content of the review, wrote the abstract and the title, coordinated the work of the authors and corrected all paragraphs. All authors edited the text and commented on the figures.
